# Supplementary material for: Diagnosis, clinical features, and mortality risk factors in a Chinese cohort with pulmonary mucormycosis
Source: PLoS One. 2025 May 16;20(5):e0323624. doi: 10.1371/journal.pone.0323624 (PMC12083791; doi:10.1371/journal.pone.0323624)
Supplement: Attachment — Submitted filename: Response_to_Reviewers_auresp_2.docx [file pone.0323624.s003.docx]

Esteemed Editors and Reviewers,

We extend our heartfelt gratitude for your thorough review and invaluable feedback on our manuscript titled "Diagnosis, Clinical Features, and Mortality Risk Factors in a Chinese Cohort with Pulmonary Mucormycosis" (Manuscript Number: PONE-D-24-59709). In accordance with the suggestions provided by the reviewers and editors, we have meticulously revised our manuscript. Herein, we address each of the reviewers' comments point by point. It is our sincere hope that these revisions meet the journal's standards and contribute to the enhancement of the manuscript's overall quality.

1. Please review your reference list to ensure that it is complete and correct. If you have cited papers that have been retracted, please include the rationale for doing so in the manuscript text, or remove these references and replace them with relevant current references. Any changes to the reference list should be mentioned in the rebuttal letter that accompanies your revised manuscript. If you need to cite a retracted article, indicate the article’s retracted status in the References list and also include a citation and full reference for the retraction notice.

**Re: We have reviewed the reference list to ensure its completeness and accuracy. All cited references are up-to-date and have not been retracted.**

2. CHECK THE SPELLING OF MUCORMYCOSIS --THROUGH OUT THE MANUSCRIPT

**Re: We have thoroughly checked the manuscript and corrected the spelling of "mucormycosis" throughout. It is now consistent and accurate.**

3. DEFINE COVID-19 IN FULL IN FIRST USE

**Re: We have defined "COVID-19" in full as "Coronavirus Disease 2019" at its first mention in the manuscript, as per your request.**

4. DEFINE HR AND CI IN THE ABSTRACT IN THE FIRST

**Re: We have defined "HR" (Hazard Ratio) and "CI" (Confidence Interval) in full at their first occurrence in the abstract.**

**We believe that these revisions have adequately addressed the comments from the reviewers and editors, significantly enhancing the quality of the manuscript. We look forward to your further feedback.**
